# State-level public health preparedness indices as predictors of COVID-19 mortality outcomes: results from the United States of America in 2020

**DOI:** 10.3389/fepid.2023.1229718

**Published:** 2023-12-12

**Authors:** Matthew R. Boyce

**Affiliations:** Center for Global Health Science & Security, Georgetown University, Washington, DC, United States

**Keywords:** assessment, COVID-19, health security, mortality, preparedness, public health

## Abstract

This study evaluates associations between state-level preparedness indices and reported COVID-19-related mortality outcomes in all 50 states and the District of Columbia in the United States of America during three distinct time periods throughout the first year of the COVID-19 pandemic. State-level preparedness data for the year 2019 were gathered from the National Health Security Preparedness and Trust for America's Health Indices, and COVID-19-related mortality data for March–December 2020 (i.e., excess mortality and reported COVID-19 mortality rates) were collected in May 2022. Linear regression analyses were conducted to examine associations during three distinct time periods. Statistically significant positive associations were observed between both indices and reported COVID-19 mortality rates during the first time period. A statistically significant negative association was observed between one preparedness index and excess mortality during the second time period. No other significant associations existed for the outcomes or time periods considered in this analysis. These results demonstrate that state-level preparedness indices were not well attuned to COVID-19-related mortality outcomes during the first year of the pandemic. This suggests that current measures of state-level preparedness may be underinclusive and require a reconceptualization to improve their utility for public health practice.

## 1. Introduction

Prior to the Coronavirus Disease 2019 (COVID-19) pandemic, there had been several ongoing efforts in the United States of America (U.S.) to prepare for such an event and assess how ready the country was to respond to a public health emergency. Since 2010 and under the Monitoring and Evaluation Framework for the International Health Regulations, the country has completed annual self-assessments of the capacities required to prevent, detect, and respond to public health emergencies. The U.S. also completed a joint external evaluation in 2016 and developed a national action plan for health security in 2018 (1). The aforementioned national-level assessments generally reported the country to be among the most prepared, if not the most prepared, in the world.

However, in addition to these well-known national-level assessments, a number of non-governmental, state-level assessments also exist. These include the Trust for America's Health State Public Health Preparedness Index (TFAHI) and the National Health Security Preparedness Index (NHSPI). These efforts are complementary projects that work in combination to measure and improve the country's health security and emergency preparedness (2).

The TFAHI has tracked public health emergency preparedness in the United States since 2003 for events including infectious disease outbreaks, natural disasters, and bioterrorism (2). This index is informed by a variety of measures that have evolved over time, but recent iterations have assessed the country's level of preparedness on a state-by-state basis using 10 priority indicators: incident management, cross-sector community collaboration, public health accreditation boards, emergency management accreditation boards, state public health funding, water security, workforce resilience and infection control, countermeasure utilization, patient safety, and health security surveillance (2).

The NHSPI represents another assessment that has measured state preparedness since 2013. Similarly, the specific methodology used to assess preparedness has evolved over time, but recent iterations contained 129 measures of various capabilities that are important for protecting against the health consequences of large-scale hazards and emergencies (3). These measures are organized across six broad domains: health security surveillance, community planning and engagement, information and incident management, healthcare delivery, countermeasure management, and environmental and occupational health (3).

However, while these efforts to assess emergency preparedness are valuable for priority setting and monitoring capacities, ultimately, their value is diminished unless demonstrated to be associated with outcomes in the response to actual public health emergencies. In this context, there have been ongoing inquiries into the validity of these preparedness assessments and how well they have been aligned with outcomes throughout the pandemic. At the national-level, previous work has shown that preparedness assessments (i.e., the joint external evaluation) were not highly correlated with COVID-19-related mortality outcomes (4, 5). This, naturally, begets the question of whether state-level preparedness assessments were predictive of COVID-19 outcomes. Previous work investigating this question has suggested that NHSPI assessment scores were not valid predictors of excess mortality six months after the pandemic had begun (6).

Although these results are important, the analyses were published within a window where prior work has shown that the provisional mortality data used to calculate excess mortality are incomplete (7), and others have written about how these analyses may have been conducted prematurely (8). Furthermore, other research has shown that, in the U.S., the COVID-19 pandemic was defined by three periods in 2020—the initial surge that occurred from March through May, the flattening of the curve from June through September, and a winter surge from October through December (9). Importantly, the extent to which state public health systems could reasonably be expected to respond well to the pandemic might vary according to the period of time considered. This is especially true as less was known about effective response measures early in the pandemic and some response capabilities, such as vaccine distribution, were not relevant until later in the outbreak.

As such, there is a need to investigate other indices (e.g., the TFAHI) and different time periods now that more time has passed and the data are more complete—as associations between preparedness assessments and COVID-19 outcomes are likely to differ depending on the quality of the data, as well as the time interval and preparedness assessment considered. The objectives of this analysis, then, are to investigate if preparedness as measured by the NHSPI and TFAHI was a valid predictor of states' COVID-19 mortality outcomes at different time periods throughout the first year of the pandemic, prior to vaccine distribution to the public.

## 2. Methods

This analysis used a series of linear regressions to investigate the relationship between state-level preparedness data and COVID-19 mortality outcomes in all 50 states and the District of Columbia in the United States of America. As defined by previous research, the time periods considered included Period 1 (i.e., March 1–May 30, 2020), Period 2 (i.e., May 31–October 3, 2020), and Period 3 (i.e., October 4–December 26, 2020) (9). The primary hypothesis for this work was that higher preparedness scores would be associated with better COVID-19 mortality outcomes (i.e., less death).

### 2.1. Data collection

Data collection occurred in May 2022. The independent variables for this analysis were 2019 state-level preparedness as measured by the TFAHI and the NHSPI, respectively. Preparedness data from 2019 were chosen because they represent the most valid data for the time at which the outbreak began (i.e., in late 2019).

The 2019 TFAHI data were sourced from the 2020 edition of the Ready or Not: Protecting the Public's Health from Diseases, Disasters, and Bioterrorism Issue Report (2). This report does not explicitly provide the preparedness scores, but rather groups states and the District of Columbia into three ordinal tiers—high, middle, and low—based on their performances across the 10 indicators used to assess preparedness. However, the report includes a detailed methodology in an appendix that allows researchers to calculate preparedness scores based on data published in the report. This methodology was used to calculate the preparedness scores for each state and the District of Columbia, which were subsequently used in the analysis. The 2019 preparedness data as measured by the NHSPI were sourced from the 2020 release of the NHSPI (3).

The dependent variables for this analysis were the excess mortality rate per 100,000 population and the reported COVID-19 mortality rate per 100,000 population in the specified time periods. Both variables were used in the analysis because COVID-19 mortality data are prone to biases that may arise from a variety of factors including differences in case definitions, reporting practices, public health capacity, and attitudes (9, 10). Excess mortality estimates represent one method for avoiding this potential bias and addressing systematic differences in the identification and reporting of COVID-19 mortality (9–11). Thus, for this analysis, excess mortality rates were considered to be the primary outcome measure and reported COVID-19 mortality a secondary outcome measure.

Excess mortality data and reported COVID-19 mortality count data were retrieved from the United States Centers for Disease Control and Prevention (CDC) National Center for Health Statistics (12). Excess mortality, as defined by the CDC, is the difference between the observed number of deaths occurring in a specific period of time and the number of deaths expected to occur in the same period. Estimates of expected deaths were calculated by the CDC using Farrington surveillance algorithms, which use over-dispersed Poisson generalized linear models with spline terms to model trends in death counts while accounting for seasonality (13). Mortality rates were then calculated as the number of deaths per 100,000 persons in each state and the District of Columbia using 2020 population data gathered from the United States Census (14).

### 2.2. Data analysis

Data were compiled in a Microsoft Excel spreadsheet (Redmond, WA) and data analyses were conducted using STATA v.17BE (College Station, TX). Basic descriptive statistics were calculated for the independent and dependent variables. Pearson correlation tests were performed to investigate if state-level preparedness assessments, as measured by the two different indices, were correlated. Linear regression models were then used to investigate the associations between preparedness, as measured by the NHSPI and TFAHI, and states' COVID-19-related mortality outcomes during the three time periods.

## 3. Results

The mean TFAHI state-level preparedness score was 5.54 (range, 3.25–7.00) and the mean NHSPI score was 6.76 (range, 5.70–7.50). Pearson's correlation test results showed that there was a moderate, positive correlation (*r *= 0.63, *p* = 0.000) between the two preparedness indices, indicating a consensus regarding state-level preparedness. The mean state-level excess mortality rate was 27.59 deaths per 100,000 population (SD, 35.10) during the first period, 38.46 deaths per 100,000 population (SD, 24.95) during the second period, and 60.59 deaths per 100,000 population (SD, 32.53) during the third period (Table 1); The median state-level COVID-19 mortality rate was 14.15 deaths per 100,000 population during the first period (IQR, 5.67–33.91), 32.43 deaths per 100,000 population during the second period (IQR, 23.32–45.60), and 58.24 deaths per 100,000 population during the third period (IQR, 34.25–100.86).

**Table 1:** Summary statistics for COVID-19-related mortality rates per 100,000 population (*n* = 51).

Measure	Period	Median (IQR)	Mean (SD)	Range
EM	Period 1 (Mar 1–May 30)	14.15 (5.67–33.91)	27.59 (35.10)	1.20–181.05
	Period 2 (May 31–Oct 3)	32.43 (23.32–45.60)	38.46 (24.95)	2.85–112.85
	Period 3 (Oct 4–Dec 26)	58.24 (34.25–100.86)	60.59 (32.53)	7.18–154.53
C19	Period 1 (Mar 1–May 30)	13.86 (6.12–33.03)	25.71 (31.25)	0.00–146.57
	Period 2 (May 31–Oct 3)	21.36 (12.62–29.73)	24.94 (17.74)	0.00–77.87
	Period 3 (Oct 4–Dec 26)	54.04 (29.90–78.36)	55.08 (31.32)	5.35–153.34

EM, excess mortality rate; C19, reported COVID-19 mortality rate.

**Figure 1:**
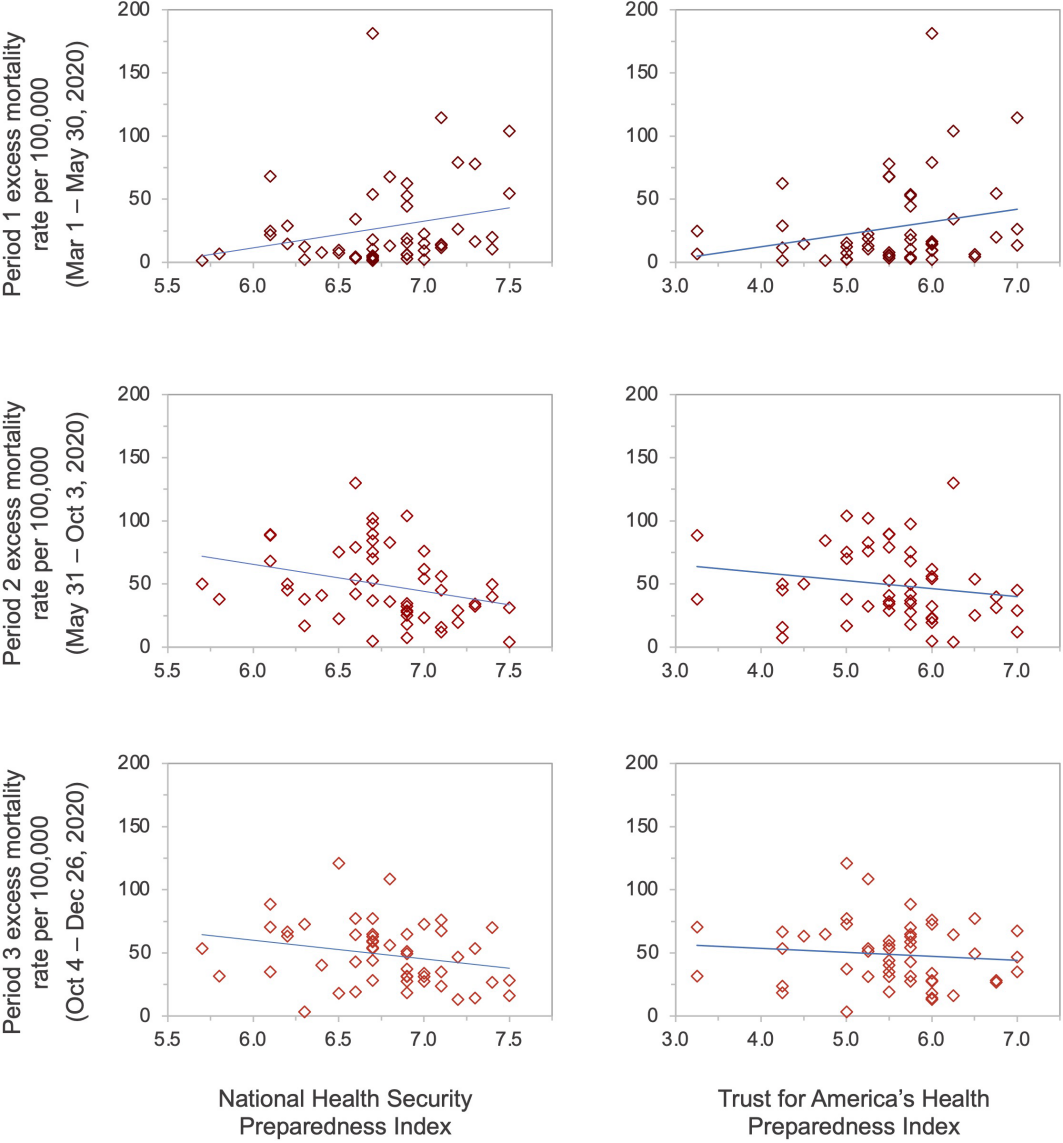
State-level preparedness as measured by the National Health Security Preparedness Index and the Trust for America's Health Index versus excess mortality rates per 100,000 population.

A positive association existed between higher preparedness as measured by the NHSPI and excess mortality during the first period (*β* = 21.14, *p* = 0.078), and negative associations existed during the second (*β* = −18.52, *p* = 0.029) and third periods (*β* = −16.83, *p* = 0.132) (Table 2; Figure 1); the associations during the first and third periods were not statistically significant. Similarly, there was a positive association between higher preparedness as measured by the TFAHI and excess mortality during the first period (*β* = 9.91, *p* = 0.096), with negative associations existing during the second (*β* = −4.28, *p* = 0.315) and third time periods (*β* = −3.81, *p* = 0.494); none of the associations between preparedness as measured by the TFAHI and excess mortality were statistically significant.

**Table 2:** Associations between COVID-19-related mortality rates per 100,000 population and state-level preparedness indices.

Measure	Preparedness index	Period 1 (Mar 1–May 30)	Period 2 (May 31–Oct 3)	Period 3 (Oct 4–Dec 26)
		β (95% CI)	β (95% CI)	β (95% CI)
EM	NHSPI	21.14 (−2.47, 44.76)	−18.52 (−35.02, −2.02)*	−16.83 (−38.91, 5.25)
	TFAHI	9.91 (−1.81, 21.63)	−4.28 (−12.77, 4.20)	−3.81 (−14.93, 7.31)
C19	NHSPI	21.65 (0.84, 42.45)*	−6.11 (−18.31, 6.09)	−9.64 (−31.22, 11.94)
	TFAHI	10.73 (0.44, 21.01)*	1.55 (−4.52, 7.63)	−0.85 (−11.61, 9.90)

EM, excess mortality rate; C19, reported COVID-19 mortality rate.

* Denotes *p* < 0.05.

**Figure 2:**
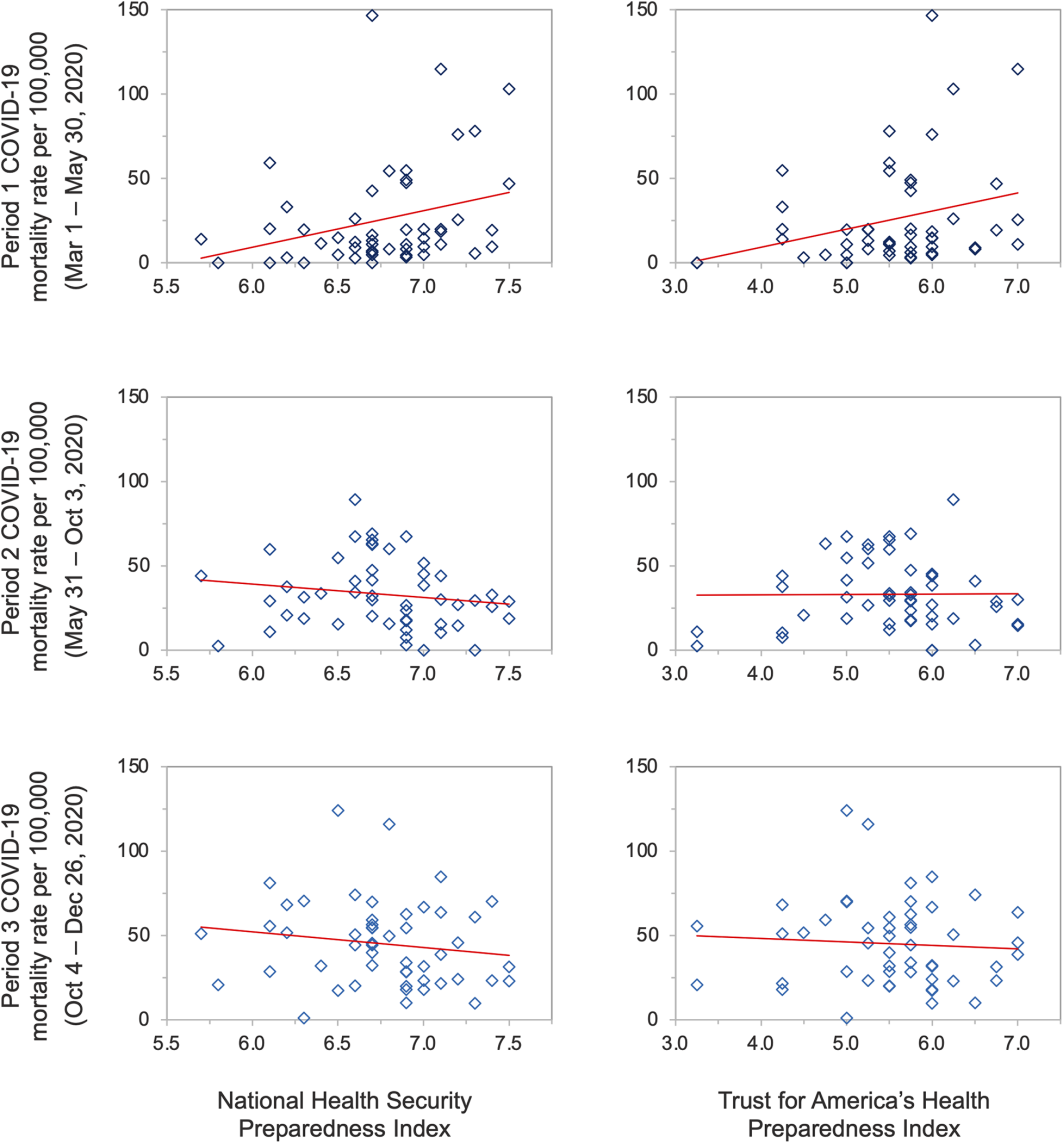
State-level preparedness as measured by the National Health Security Preparedness Index and the Trust for America's Health Index versus reported COVID-19 mortality rates per 100,000 population.

The mean state-level COVID-19 mortality rate was 25.71 deaths per 100,000 population (SD, 31.25) during the first period, 24.94 deaths per 100,000 population (SD, 17.74) during the second period, and 55.08 deaths per 100,000 population (SD, 31.32) during the third period (Table 1); The median state-level COVID-19 mortality rate was 13.86 deaths per 100,000 population during the first period (IQR, 6.12–33.03), 21.36 deaths per 100,000 population during the second period (IQR, 12.62–29.73), and 54.04 deaths per 100,000 population during the third period (IQR, 29.90–78.36). A statistically significant, positive association was observed between higher NHSPI preparedness and COVID-19 mortality rates during the first period (*β* = 21.65, *p* = 0.042), and non-significant, negative associations during the second (*β* = −6.11, *p* = 0.319) and third periods (*β* = −9.64, *p* = 0.374) (Table 1; Figure 2). A statistically significant positive association also existed between higher preparedness as measured by the TFAHI and excess mortality during the first period (*β* = 10.73, *p* = 0.041), as well as during the second period (*β* = 1.55, *p* = 0.610), with a negative association during the third period (*β* = −0.85, *p* = 0.874); the associations during the second and third periods were not statistically significant.

## 4. Discussion

This analysis sought to investigate the associations between state-level preparedness as measured by the NHSPI and TFAHI and COVID-19-related mortality outcomes throughout the first year of the pandemic. Contrary to what was hypothesized, the results suggest that higher preparedness was associated with higher excess mortality rates and higher reported COVID-19 mortality rates early in the pandemic (i.e., during Period 1), with statistically significant associations existing between preparedness scores and COVID-19 mortality rates. Still, during other periods of time (i.e., Periods 2 and 3), the hypothesis was correct and negative associations existed between preparedness and COVID-19-related mortality outcomes, with a statistically significant relationship existing between excess mortality rates during Period 2 and preparedness as measured by the NHSPI.

This analysis adds to a growing evidence base investigating the relationship between measures of public health preparedness and mortality outcomes throughout the COVID-19 pandemic. Previous work has suggested that existing measures of preparedness were not well attuned to mortality outcomes throughout the pandemic (4–6). While the results from this analysis broadly agree with these conclusions and suggest that measures of preparedness were not significantly associated with pandemic mortality outcomes throughout much of the first year, a significant, negative association was observed between the NHSPI and excess mortality during the second period. This result suggests that the relationship between preparedness measures and pandemic outcomes may be more nuanced than initially believed.

Indeed, key enabling or disabling factors may exist that determine whether public health preparedness capacity translates to effective public health response. A single political decision, for example, could determine both if and when public health capacities are used during the response to an infectious disease outbreak. This political action or inaction holds the potential to influence downstream effects and health outcomes, such as mortality (15). Furthermore, the period of time considered may hold important implications for the relative importance of preparedness capacities. Early in the pandemic, when blunt public health tools such as lockdowns were being widely used, public health preparedness capacities may have held relatively less importance. Still, during later periods, when society began to reopen, preparedness capacities may have been more important in mitigating the negative health consequences of the pandemic.

Relatedly, it is important to emphasize that these results represent predictive associations and that higher levels of preparedness are unlikely to have caused higher levels of COVID-19 mortality early in the pandemic. In fact, there is an argument to be made that those states assessed as being more relatively prepared are likely to have better surveillance and reporting systems, resulting in the reporting of more fatalities and surveillance bias (16). What seems more probable is that states reporting higher levels of preparedness were simply impacted by the pandemic first when less was known about the virus, resulting in higher levels of mortality. Previous work has demonstrated that states in the Midwest and Northeast were disproportionately affected earlier in the pandemic compared to states in the South and/or West (9); many states in these regions were among those assessed as better prepared, with Connecticut, Iowa, Kansas, Maine, Massachusetts, Missouri, Nebraska, Pennsylvania, Vermont, and Wisconsin and all reporting above-average preparedness in both the NHSPI and TFAHI.

These results must be interpreted in a fashion that acknowledges their limitations. As discussed, the surveillance data that represent the dependent variable are prone to both biases and confounding; and COVID-19 mortality data—while representing the most direct mortality outcome associated with the pandemic—are particularly prone to both reporting and surveillance biases. Finally, excess mortality estimates depend on provisional data that are often incomplete. While previous work has noted that these data are generally greater than 99 percent complete by 39 weeks following a death (7)—and this work has been conducted well after that time period—at the time of writing, the CDC still reports lagged death counts and notes that death counts for some states in 2020 may be underestimated (12).

Future research should continue to examine the relationship between preparedness measures and COVID-19 mortality outcomes as the validity of COVID-19 mortality data improves and we better understand the factors that impact mortality. As these factors are better understood, questions of causality may be investigated. Future research may also wish to investigate the relationship between state-level preparedness measures and other key pandemic response metrics, such as the time required to control the spread of COVID-19, the number of reported cases, or vaccination uptake.

This study showed that higher state-level preparedness scores were associated with significantly higher levels of COVID-19-related mortality early in the pandemic (i.e., Period 1) but lower COVID-19-related mortality later in the pandemic (i.e., Periods 2 and 3). These results hold important implications for both public health research and practice. The implications for public health research relate to work focused on the COVID-19 pandemic, specifically efforts that consider or use COVID-19 mortality outcomes. Many have cautioned against using COVID-19 mortality data in research and promoted the use of excess mortality associated with COVID-19 instead (9–11). This analysis advances this stance by providing a concrete example and proof-of-concept of how the results and subsequent policy implications can differ depending on which mortality outcome is used. Future research that uses COVID-19 mortality outcomes should, therefore, account for these considerations.

Beyond this, the implications of this research for public health practice—and perhaps the most significant implications of this research—are that current measures of state-level preparedness require a reconceptualization. While higher measures of preparedness were negatively associated with COVID-19-related mortality outcomes during Period 2 and Period 3 of the first year of the pandemic, only one of these associations was statistically significant. As previously noted, public health assessments that measure preparedness are crucially important for setting priorities and tracking progress over time but there is limited utility for preparedness indices that have no proven associations with actual outcomes during public health emergencies (6). There is increasing recognition of political determinants of health and discussions surrounding national-level preparedness measures have emphasized the need to better incorporate aspects such as health system fragmentation, political leadership, socioeconomic inequalities, and trust in government (5, 15, 17). Further, other work has demonstrated that sociopolitical and governance variables—including social cohesion, social polarization, and perceived corruption—were correlated with reduced excess mortality throughout the COVID-19 pandemic (18). Given that many of these aspects are not well captured in the NHSPI or TFAHI, incorporating these attributes into preparedness assessments may represent a reasonable starting point for reconceptualizing and revising state-level preparedness indices. Doing so could help to ensure that the experiences from COVID-19 are used to better prepare the United States for future public health emergencies.

## Data Availability

Publicly available datasets were analyzed in this study. This data can be found here: National Health Security Preparedness Index. Toolkit: 2020 Archives. https://nhspi.org/tools-resources/. Trust for America's Health. Ready or Not: Protecting the Public's Health from Diseases, Disasters, and Bioterrorism—2020 Issue Report. https://www.tfah.org/report-details/readyornot2020/. National Center for Health Statistics. COVID-19 Data from the National Center for Health Statistics. https://www.cdc.gov/nchs/covid19/index.htm. United States Census Bureau. 2020 US Census Bureau Data. https://data.census.gov/cedsci/.
